# Denosumab versus zoledronic acid for primary osteoporosis: a 36-month retrospective cohort study on bone mineral density changes

**DOI:** 10.3389/fmed.2026.1842793

**Published:** 2026-07-29

**Authors:** Xin Zou, Xiaomeng Huang, Mingxin Tang, Yuyan Zhu, Cheng Li, Liangjun Zhao

**Affiliations:** 1 Department of Bone and Joint Surgery, The First Affiliated Hospital of Guangxi Medical University, Nanning, Guangxi, China; 2 Department of Bone and Joint Surgery, The First Affiliated Hospital of Guangxi University of Science and Technology, Guangxi University of Science and Technology, Liuzhou, Guangxi, China; 3 School of Automation, Guangxi University of Science and Technology, Liuzhou, Guangxi, China; 4 Department of Geriatrics, Hezhou Guangji Hospital, Hezhou, Guangxi, China

**Keywords:** bone mineral density, bone turnover markers, denosumab, primary osteoporosis, zoledronic acid

## Abstract

**Objective:**

To explore and compare the clinical efficacy and safety of denosumab versus zoledronic acid for the treatment of primary osteoporosis.

**Methods:**

This retrospective cohort study included 413 primary osteoporosis patients (denosumab, *n* = 198; zoledronic acid, *n* = 215). All received daily calcium (600 mg) and vitamin D (400 IU), representing the total combined daily intake from diet and supplements. The denosumab group received subcutaneous denosumab 60 mg every 6 months and the zoledronic acid group annual intravenous zoledronic acid 5 mg over 36 months. Outcomes assessed at baseline and after 36 months included bone mineral density (BMD) at the lumbar spine, femoral neck, and total hip; bone turnover markers β-CTX (β-isomerized C-terminal telopeptide of type I collagen) and P1NP (procollagen type I N-terminal propeptide); pain severity using the Visual Analog Scale (VAS); functional impairment using the Oswestry Disability Index (ODI); and incidence of adverse reactions.

**Results:**

After 36 months, BMD at all sites significantly increased from baseline in both groups (*P* < 0.001). The denosumab group demonstrated greater mean absolute increases in BMD at the lumbar spine (0.13 ± 0.10 vs. 0.10 ± 0.10 g/cm^2^, *P* < 0.001), femoral neck (0.14 ± 0.06 vs. 0.12 ± 0.05 g/cm^2^, *P* < 0.001), and total hip (0.09 ± 0.12 vs. 0.05 ± 0.09 g/cm^2^, *P* < 0.001) compared to the zoledronic acid group. Both groups showed significant decreases in β-CTX and P1NP levels (*P* < 0.001), with the denosumab group exhibiting a greater reduction in β-CTX (−0.54 ± 0.47 vs. −0.34 ± 0.44 ng/mL, *P* < 0.001) and P1NP (−23.68 ± 5.44 vs. −19.98 ± 5.28 ng/mL, *P* < 0.001). VAS and ODI scores improved significantly in both groups (*P* < 0.001), with a greater reduction in the VAS score in the denosumab group (−5.17 ± 1.58 vs. −4.61 ± 1.48, *P* < 0.001). The overall incidence of adverse reactions was lower in the denosumab group compared to the zoledronic acid group (5.56% vs. 20.00%, *P* = 0.008). Adverse events were collected via passive EMR retrieval (see Methods).

**Conclusion:**

Over 36 months, both agents were effective. Denosumab was associated with greater BMD gains, stronger bone resorption suppression, and fewer recorded adverse reactions. These findings are hypothesis-generating and await prospective validation.

## 1 Introduction

Primary osteoporosis is a systemic metabolic bone disease characterized by progressive bone loss and microarchitectural deterioration of bone tissue. Its pathophysiology involves an imbalance in bone remodeling, where osteoclast-mediated bone resorption exceeds osteoblast-mediated bone formation, leading to increased bone fragility and fracture risk (1). Fragility fractures, commonly occurring at the spine, hip, distal forearm, and proximal humerus, represent a major cause of morbidity, including acute pain, functional impairment, and reduced quality of life (2, 3). In severe cases, multiple vertebral compression fractures can lead to thoracic kyphosis, cervical hyperlordosis, and associated complications such as respiratory difficulties due to diaphragmatic relaxation (4–6). Furthermore, the socioeconomic burden is substantial, with studies indicating a significantly higher risk of osteoporosis and fragility fractures in disadvantaged populations, placing considerable strain on healthcare systems and patient families (7, 8). The global prevalence of osteoporosis among older adults is estimated at 21.7%, with the highest rates observed in Asia (24.3%), underscoring the critical need for effective prevention and treatment strategies (9).

Current pharmacological interventions for osteoporosis include antiresorptive agents, bone formation promoters, and agents with dual mechanisms of action (10) antiresorptive agents are considered first-line therapy for primary osteoporosis (11). Among these, denosumab and zoledronic acid are widely used due to their distinct mechanisms and established efficacy. Denosumab, a fully human monoclonal antibody against receptor activator of nuclear factor kappa-B ligand (RANKL), binds to RANKL, preventing its interaction with RANK on osteoclast precursors and mature osteoclasts. This inhibits osteoclast differentiation, activation, and survival, thereby reducing bone resorption and increasing bone mineral density (BMD) (12, 13). Zoledronic acid, a third-generation bisphosphonate, exhibits high affinity for hydroxyapatite in bone and is taken up by osteoclasts during bone resorption. It inhibits farnesyl pyrophosphate synthase, disrupting osteoclast function and inducing apoptosis, ultimately suppressing bone resorption (13–15). Current evidence suggests that denosumab may offer advantages over zoledronic acid in treating primary osteoporosis, including greater increases in lumbar spine and total hip BMD, pain relief, and a lower incidence of adverse reactions such as myalgia, fever, and gastrointestinal issues (16, 17).

While numerous studies have confirmed the efficacy of both agents in increasing BMD and reducing fracture risk (18), there is a relative paucity of long-term, head-to-head comparative studies directly evaluating their effectiveness in the Chinese population. Specifically, data from studies systematically assessing changes in BMD at multiple skeletal sites, bone turnover markers (BTMs), and patient-reported outcomes over a 3-year period remain limited. To address this evidence gap, we conducted a 36-month retrospective analysis to systematically compare the clinical efficacy and safety of denosumab (*n* = 198) and zoledronic acid (*n* = 215). Both agents are commonly used long-acting anti-osteoporosis treatments in clinical practice, offering convenient administration and broad applicability. Given their substantial differences in mechanisms of action, acute-phase reaction profiles, and long-term safety considerations, real-world comparative data are of high clinical value for guiding treatment decisions in patients with primary osteoporosis. This study aims to provide evidence-based support for individualized treatment decisions by comprehensively evaluating key outcomes, including BMD, BTMs (β-CTX, P1NP), pain scores (VAS), functional disability (ODI), adverse events, and fracture incidence.

## 2 Materials and methods

### 2.1 Study design and population

This single-center, retrospective cohort study included patients diagnosed with osteoporosis at the First Affiliated Hospital of Guangxi University of Science and Technology between January 2021 and January 2023, patients were enrolled between January 2021 and January 2023. The final follow-up assessment was conducted in January 2026. For patients enrolled in January 2023, the 36-month follow-up was completed by January 2026; therefore, the January 2026 assessment represents the last available follow-up time point for the entire cohort, with all patients having completed at least 36 months of treatment by the time of data extraction. Inclusion criteria were: (1) diagnosis of primary osteoporosis according to established criteria, confirmed by dual-energy X-ray absorptiometry (DXA) with a T-score ≤−2.5 and/or a history of fragility fractures (1); (2) age ≥ 50 years; (3) no prior anti-osteoporosis medication within the preceding year; and (4) completion of a full 36-month treatment course with either denosumab or zoledronic acid; patients who switched treatment regimens during follow-up were excluded. Exclusion criteria were: (1) secondary osteoporosis (e.g., due to hyperthyroidism, rheumatoid arthritis, malignancy, metabolic bone disease, autoimmune disorders, a history of multiple myeloma, or glucocorticoid use >3 months); (2) severe hepatic or renal impairment (serum creatinine ≥ 400 μmol/L or eGFR < 30 mL/min); (3) pregnancy or lactation; (4) conditions potentially affecting treatment efficacy, such as hypocalcemia; (5) incomplete follow-up records or medical records; (6) switching of treatment regimens during the follow-up period. All patients underwent screening for serum phosphorus, parathyroid hormone, 25-hydroxyvitamin D, and multiple myeloma-related workup to comprehensively exclude secondary causes of bone disease. For patients found to have vitamin D deficiency at screening (25-hydroxyvitamin *D* < 20 ng/mL), vitamin D repletion was initiated prior to starting anti-osteoporosis therapy. The study was approved by the institutional ethics committee, and the requirement for informed consent was waived due to the retrospective design. All patient data were de-identified to ensure privacy. All findings from this analysis are considered exploratory and are intended to inform the design of future confirmatory studies, rather than to provide definitive clinical guidance.

### 2.2 Treatment protocol

Patients were divided into two groups based on the treatment received: a denosumab group (*n* = 198) and a zoledronic acid group (*n* = 215). All patients received basic treatment comprising daily oral supplementation with 600 mg elemental calcium and 400 IU vitamin D, representing the total combined daily intake from diet and supplements. All patients received osteoporosis education and fall prevention guidance.

Denosumab Group: Patients received subcutaneous injections of denosumab 60 mg (administered in the anterior thigh, abdomen, or upper arm) every 6 months for a total of 6 doses over 36 months.

Zoledronic Acid Group: Patients received annual intravenous infusions of zoledronic acid 5 mg, diluted in 100 mL of 0.9% sodium chloride and infused over at least 15 min, for a total of 3 doses over 36 months. To prevent acute phase reactions, patients received oral acetaminophen 0.5 g 30 min prior to each infusion.

### 2.3 Medication adherence assessment

Medication adherence was systematically assessed throughout the 36-month treatment period. For patients in the denosumab group, adherence was evaluated by documenting the date of each subcutaneous injection (administered every 6 months). For patients in the zoledronic acid group, adherence was evaluated by documenting the date of each intravenous infusion (administered annually). All injection and infusion dates were extracted from the hospital’s electronic medical record system and cross-referenced with the prescribed dosing schedule.

Adherence was classified according to the following criteria:

Full adherence: All scheduled doses are administered within the prescribed time window (denosumab: ± 4 weeks of the 6-month interval; zoledronic acid: ± 4 weeks of the 12-month interval).
Delayed administration: Doses administered beyond the ± 4-week window but within 8 weeks of the scheduled date.
Missed dose: Any scheduled dose not administered by 8 weeks after the scheduled date.
Treatment completion rate: The proportion of patients who completed all scheduled doses (6 doses for denosumab, 3 doses for zoledronic acid) within the 36-month period.

### 2.4 Outcome measures

Data were extracted from the hospital’s electronic medical record system. The post-treatment assessment was based on the last follow-up examination, conducted in January 2026. All outcome measurements were performed at baseline and after 36 months of treatment.

Bone Mineral Density (BMD): BMD (g/cm^2^) at the lumbar spine (L1–L4), femoral neck, and total hip was measured using dual-energy X-ray absorptiometry (DXA) (Hologic Discovery A, Hologic Inc., Bedford, MA, USA) before and after treatment. All DXA scans were performed by trained technicians following a standardized positioning protocol. Daily quality assurance was performed using a hydroxyapatite phantom, and the coefficient of variation for the DXA machine was <1.0% for lumbar spine and <1.5% for femoral neck measurements during the study period. The least significant change (LSC) for BMD was 0.015 g/cm^2^ for the lumbar spine and 0.020 g/cm^2^ for the femoral neck, as determined by repeat measurements in 15 healthy volunteers. Inter- and intra-observer reproducibility was maintained by having all scans reviewed by a single certified radiologist. BMD changes are reported as both absolute changes (g/cm^2^) and percentage changes from baseline. T-scores were also recorded, with osteoporosis defined as a T-score ≤−2.5 (1).

Bone Turnover Markers (BTMs): Fasting venous blood samples (5 mL) were collected before and after 36 months of treatment. Serum levels of β-isomerized C-terminal telopeptide of type I collagen (β-CTX) and procollagen type I N-terminal propeptide (P1NP) were measured using enzyme-linked immunosorbent assay (ELISA) according to the manufacturers’ instructions.

Pain and Functional Impairment: Pain intensity was assessed using the Visual Analog Scale (VAS), ranging from 0 (no pain) to 10 (worst imaginable pain). VAS scores primarily assess osteoporosis-related bone pain in the lumbosacral spine and hip regions, including both resting pain and pain exacerbated by activity, rather than pain strictly attributable to acute fractures. Functional disability was evaluated using the Oswestry Disability Index (ODI) questionnaire, which assesses limitations in ten activities of daily living. Higher ODI scores indicate greater functional impairment.

Safety: Adverse events were collected via passive retrieval from electronic medical records. All adverse events occurring during the treatment period were recorded, including fever, myalgia, bone pain, dizziness, gastrointestinal reactions, and pruritus. Adverse events were counted per patient (i.e., a patient with multiple events was counted once). The incidence and severity of adverse reactions were documented. Severe adverse events necessitated treatment discontinuation and appropriate management. All new osteoporotic fractures during the treatment period were also recorded, with fractures identified through telephone and outpatient follow-up based on patient self-report.

### 2.5 Statistical analysis

Statistical analysis was performed using SPSS version 27.0. Normality of continuous variables was assessed using the Shapiro-Wilk test. Continuous data are presented as the mean ± standard deviation (SD). Within-group comparisons before and after treatment were analyzed using paired *t*-tests for normally distributed data or Wilcoxon signed-rank tests for non-normally distributed data. Between-group comparisons of continuous variables were analyzed using independent-samples *t*-tests or Mann-Whitney U tests as appropriate. Categorical data are presented as frequencies and percentages, and between-group comparisons were performed using the chi-square (χ^2^) test. A two-tailed *P*-value < 0.05 was considered statistically significant. Missing data were handled by complete-case analysis, as the proportion of missing data was small and deemed unlikely to introduce substantial bias. Given the retrospective and exploratory nature of this study, no formal sample size calculation was performed; the sample size was determined by the availability of eligible patients meeting inclusion criteria over the enrollment period. To evaluate the independent effect of treatment group assignment on 36-month changes in BMD and bone turnover markers while adjusting for potential confounders, we performed multivariable linear regression analyses. The dependent variables were the absolute changes in BMD at the lumbar spine, femoral neck, and total hip, as well as changes in β-CTX and P1NP. Independent variables included treatment group (denosumab vs. zoledronic acid), age, sex, BMI, baseline BMD at the respective site, serum calcium, serum phosphorus, eGFR, 25-hydroxyvitamin D, PTH, tobacco and alcohol use, and baseline FRAX score. Results are presented as standardized coefficients (β) and *P*-values. A two-tailed *P*-value < 0.05 was considered statistically significant. Model assumptions were assessed using residual plots and variance inflation factors to exclude multicollinearity. These analyses were exploratory and intended to generate hypotheses for future confirmatory studies.

## 3 Results

### 3.1 Search results

**FIGURE 1:**
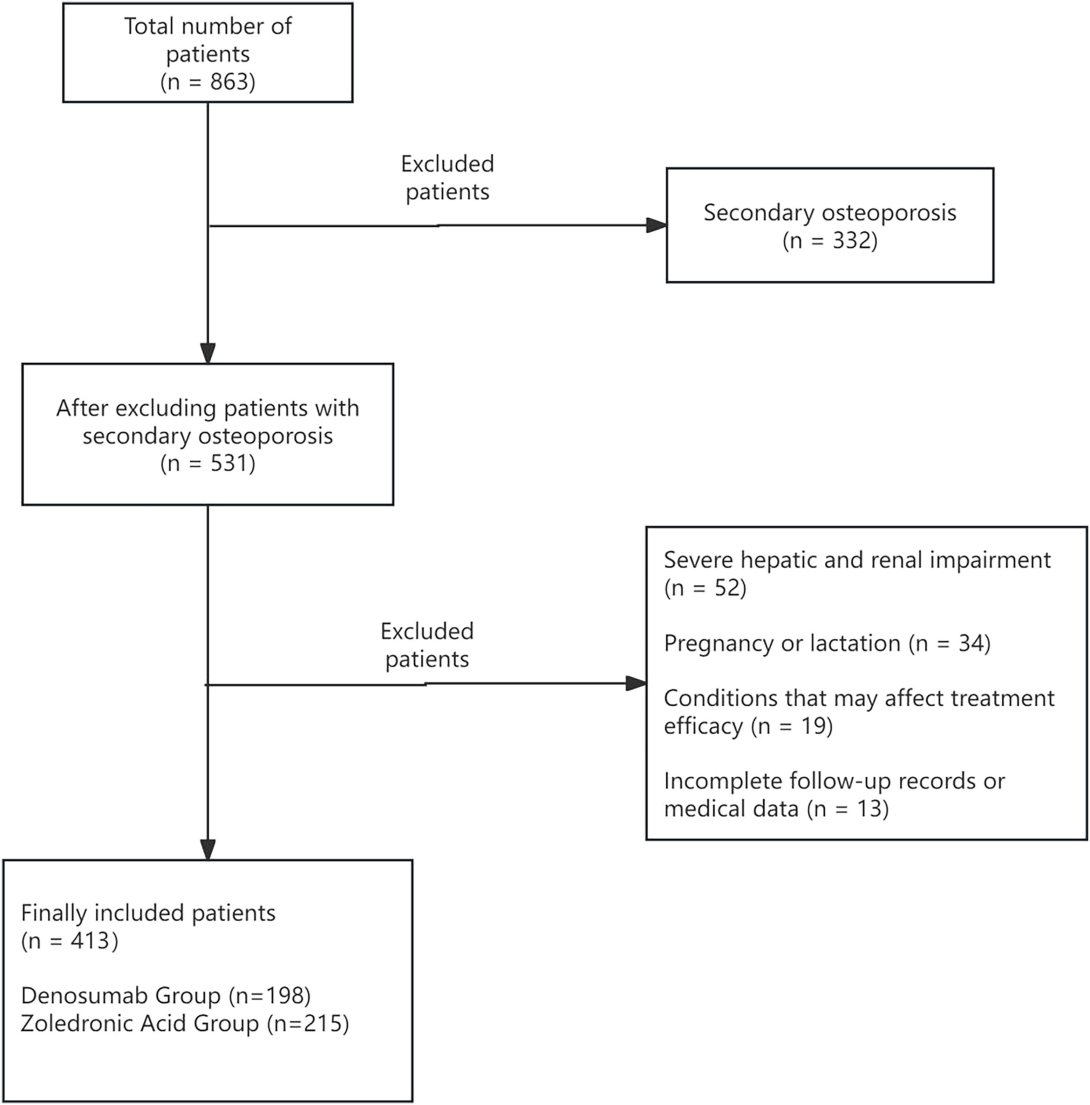
Patient selection flowchart.

The process of selecting and incorporating patients was depicted in Figure 1. A total of 863 patients with primary osteoporosis were initially assessed for eligibility. Of these, 332 patients were excluded due to a diagnosis of secondary osteoporosis, leaving 531 patients for further screening. Subsequently, 52 patients were excluded because of severe hepatic or renal impairment, 34 due to pregnancy or lactation, 19 owing to conditions that could potentially affect treatment efficacy (e.g., hypocalcemia), and 13 because of incomplete follow-up records or missing medical data. After these exclusions, 413 patients were ultimately included in the final analysis, with 198 receiving denosumab and 215 receiving zoledronic acid.

### 3.2 Medication adherence

During the 36-month follow-up, the denosumab group demonstrated a numerically higher rate of delayed administration compared with the zoledronic acid group. Specifically, 28 of 198 patients (14.1%) in the denosumab group experienced at least one delayed injection, compared with 9 of 215 patients (4.2%) in the zoledronic acid group. Missed doses occurred in 12 patients (6.1%) in the denosumab group and in 3 patients (1.4%) in the zoledronic acid group. The overall treatment completion rate was 92.4% (183/198) for the denosumab group and 97.7% (210/215) for the zoledronic acid group. The most frequently cited reasons for denosumab dose delay or missed injections included patient-initiated postponement due to scheduling conflicts (*n* = 15), travel or relocation (*n* = 10), and minor intercurrent illnesses (*n* = 8).

### 3.3 Baseline characteristics

The denosumab and zoledronic acid groups were comparable at baseline, with no significant differences in age, sex distribution, BMI, serum calcium and phosphorus levels, BMD at any site, BTMs, eGFR, 25-hydroxyvitamin D, parathyroid hormone (PTH), prevalence of prior fractures, tobacco and alcohol use, or 10-year FRAX scores (*P* > 0.05 for all), indicating that the groups were well-matched (Table 1).

**TABLE 1:** Baseline characteristics of the study population.

Characteristic	Denosumab group (*n* = 198)	Zoledronic acid group (*n* = 215)	*P*-value
Age (years)	65.08 ± 7.74	65.85 ± 8.37	0.305
Sex (male/female), (%)	93/105 (47.0/53.0%)	99/116 (46.0/54.0%)	0.772
BMI (kg/m^2^)	22.96 ± 3.43	23.20 ± 2.87	0.436
Serum calcium (mmol/L)	2.30 ± 0.53	2.30 ± 0.75	0.860
Serum phosphorus (mmol/L)	1.16 ± 0.11	1.14 ± 0.15	0.173
BMD (g/cm2)			
Lumbar spine (L1–L4)	0.72 ± 0.07	0.72 ± 0.07	0.575
Femoral neck	0.66 ± 0.03	0.65 ± 0.03	0.196
Total hip	0.67 ± 0.10	0.66 ± 0.06	0.615
β-CTX (ng/mL)	0.85 ± 0.44	0.79 ± 0.37	0.139
P1NP (ng/mL)	55.86 ± 4.40	55.31 ± 3.99	0.180
Prior fracture, *n* (%)	14 (7.1)	15 (7.0)	0.970
eGFR, (mL/min/1.73 m^2^)	68.17 ± 14.92	66.04 ± 12.19	0.112
25-OH-vitamin D, (nmol/L)	33.97 ± 9.60	33.51 ± 9.19	0.859
PTH, (pg/mL)	38.46 ± 8.99	38.41 ± 9.97	0.934
Tobacco and alcohol, *n* (%)	33%	30%	0.448
FRAX score (10-year risk of major fracture, (%)	27.84 ± 12.41	28.73 ± 12.52	0.336

Data are presented as the mean ± SD unless otherwise indicated. BMI, body mass index; BMD, bone mineral density; β-CTX, beta-isomerized C-terminal telopeptide of type I collagen; P1NP, procollagen type I N-terminal propeptide; eGFR, estimated glomerular filtration rate; PTH, parathyroid hormone; FRAX, fracture risk assessment tool.

### 3.4 Bone mineral density changes

After 36 months of treatment, BMD at all measured sites significantly increased from baseline within both groups (*P* < 0.001 for all). The denosumab group showed greater mean increases in BMD at the lumbar spine, femoral neck, and total hip compared to the zoledronic acid group (*P* < 0.001 for all comparisons) (Table 2).

**TABLE 2:** Comparison of bone mineral density changes between groups.

Site	Group	Baseline BMD (g/cm^2^)	36-month BMD (g/cm^2^)	Mean change (g/cm^2^)	Percentage change (%)	*P*-value (within group)	*P*-value (between groups)
Lumbar spine	Denosumab	0.72 ± 0.07	0.85 ± 0.09	+0.13 ± 0.10	+18.1%	<0.001	<0.001
	Zoledronic acid	0.72 ± 0.07	0.82 ± 0.08	+0.10 ± 0.10	+13.9%	<0.001	
Femoral neck	Denosumab	0.66 ± 0.03	0.79 ± 0.05	+0.14 ± 0.06	+21.2%	<0.001	<0.001
	Zoledronic acid	0.65 ± 0.03	0.77 ± 0.04	+0.12 ± 0.05	+18.5%	<0.001	
Total hip	Denosumab	0.67 ± 0.10	0.75 ± 0.08	+0.09 ± 0.12	+13.4%	<0.001	<0.001
	Zoledronic acid	0.66 ± 0.06	0.71 ± 0.07	+0.05 ± 0.09	+7.6%	<0.001	

Data are presented as mean ± SD. BMD, bone mineral density.

### 3.5 Bone turnover marker changes

Both groups demonstrated significant reductions in serum β-CTX and P1NP levels after 36 months of treatment (*P* < 0.001 for all within-group comparisons). The denosumab group exhibited a greater decrease in β-CTX compared to the zoledronic acid group (*P* < 0.001). The reduction in P1NP was also larger in the denosumab group (*P* < 0.001) (Table 3).

**TABLE 3:** Comparison of bone turnover marker changes between groups.

Marker	Group	Baseline (ng/mL)	36 months (ng/mL)	Mean change (ng/mL)	*P*-value (within group)	*P*-value (between groups)
β-CTX	Denosumab	0.85 ± 0.43	0.32 ± 0.18	−0.54 ± 0.47	<0.001	<0.001
	Zoledronic acid	0.79 ± 0.37	0.45 ± 0.27	−0.34 ± 0.44	<0.001	
P1NP	Denosumab	55.86 ± 4.40	32.18 ± 2.94	−23.68 ± 5.44	<0.001	<0.001
	Zoledronic acid	55.31 ± 3.99	35.33 ± 3.68	−19.98 ± 5.28	<0.001	

Data are presented as mean ± SD. β-CTX, beta-isomerized C-terminal telopeptide of type I collagen; P1NP, procollagen type I N-terminal propeptide.

### 3.6 Pain and functional outcomes

Visual Analog Scale and ODI scores significantly improved from baseline in both treatment groups after 36 months (*P* < 0.001). The reduction in VAS score was greater in the denosumab group compared to the zoledronic acid group (*P* < 0.001). Although both groups showed substantial improvement in ODI scores, the difference in the degree of improvement between groups was not statistically significant (*P* = 0.171) (Table 4).

**TABLE 4:** Comparison of pain and functional outcomes between groups.

Outcome	Group	Baseline	36 months	Mean change	*P*-value (within group)	*P*-value (between groups)
VAS score	Denosumab	7.54 ± 0.83	2.37 ± 1.30	−5.17 ± 1.58	<0.001	<0.001
	Zoledronic acid	7.41 ± 1.06	2.80 ± 1.14	−4.61 ± 1.48	<0.001	
ODI score	Denosumab	78.22 ± 11.47	25.73 ± 5.06	−53.97 ± 6.90	<0.001	0.171
	Zoledronic acid	76.84 ± 10.54	26.01 ± 4.34	−50.82 ± 11.42	<0.001	

Data are presented as mean ± SD. VAS, Visual Analog Scale; ODI, Oswestry Disability Index.

### 3.7 Multivariable regression analysis

Multivariable linear regression analysis was performed to identify factors independently associated with 36-month changes in BMD and bone turnover markers (Table 5). After adjusting for all baseline covariates, baseline BMD at each respective site emerged as the strongest independent determinant of the corresponding 36-month BMD outcome (all *P* < 0.001). In contrast, other covariates including age, sex, serum calcium, serum phosphorus, eGFR, 25-hydroxyvitamin D, PTH, tobacco and alcohol use, and FRAX score did not show independent associations with 36-month lumbar spine BMD changes (all *P* > 0.05). For femoral neck BMD changes, BMI showed a marginal independent association (*P* = 0.016), while tobacco and alcohol use was associated with lumbar spine BMD changes (*P* = 0.009).

**TABLE 5:** Multivariable regression analysis of factors associated with 36-month BMD and bone turnover marker changes.

Outcome	BMD	Age (years)	Sex	BMI	Serum calcium	Serum phosphorus	eGFR	25-OH-vitamin D,	PTH	Tobacco and alcohol	FRAX score	*Adjusted between-group differences (*P*-value)
Lumbar spine (L1–L4)	<0.001	0.351	0.970	0.702	0.071	0.992	0.317	0.37\9	0.636	0.009	0.456	<0.001
Femoral neck	<0.001	0.804	0.466	0.016	0.890	0.705	0.578	0.597	0.849	0.191	0.994	0.01
Total hip	<0.001	0.343	0.217	0.602	0.112	0.741	0.145	0.144	0.991	0.166	0.323	0.003
β-CTX	<0.001	0.841	0.814	0.369	0.658	0.487	0.362	0.379	0.903	0.977	0.110	<0.001
P1NP	<0.001	0.299	0.427	0.263	0.184	0.378	0.734	0.792	0.673	0.580	0.231	<0.001

Data presented are *P*-values from multivariable linear regression analyses. Each cell represents the *P*-value for the corresponding independent variable (column header) in predicting the respective 36-month outcome (row header). *Adjusted between-group differences: *P*-values for the comparison between denosumab and zoledronic acid groups, adjusted for all covariates listed in the table (age, sex, BMI, baseline BMD at the respective site, serum calcium, serum phosphorus, eGFR, 25-hydroxyvitamin D, PTH, tobacco and alcohol use, and FRAX score). BMD, bone mineral density; β-CTX, beta-isomerized C-terminal telopeptide of type I collagen; P1NP, procollagen type I N-terminal propeptide; BMI, body mass index; eGFR, estimated glomerular filtration rate; PTH, parathyroid hormone; FRAX, fracture risk assessment tool (10-year risk of major fracture).

Notably, after adjustment for all baseline covariates, treatment group assignment (denosumab vs. zoledronic acid) remained significantly and independently associated with changes in BMD at the lumbar spine, femoral neck, and total hip, as well as with changes in β-CTX and P1NP (all *P* < 0.05), indicating that the observed between-group differences were not driven by baseline imbalances.

### 3.8 Safety and fracture incidence

The overall incidence of adverse reactions was lower in the denosumab group (11/198, 5.56%) compared to the zoledronic acid group (43/215, 20.00%; *P* = 0.008). Adverse events were generally mild and resolved with symptomatic treatment; no serious adverse reactions requiring treatment discontinuation occurred in either group. In the zoledronic acid group, the most frequently reported adverse events were fever (10/215, 4.6%), dizziness (8/215, 3.7%), and gastrointestinal reactions (8/215, 3.7%), followed by myalgia (6/215, 2.7%), pruritus (6/215, 2.7%), and bone pain (5/215, 2.3%). In the denosumab group, fever was the most common event (3/198, 1.5%), followed by myalgia (2/198, 1.0%), dizziness (2/198, 1.0%), and gastrointestinal reactions (2/198, 1.0%); bone pain and pruritus were each reported in 1 patient (0.5%). The incidence rates of specific adverse events are presented in Table 6 for descriptive comparison; no conclusions regarding differential safety superiority are drawn.

**TABLE 6:** Comparison of adverse reactions and fracture incidence.

Outcome	Denosumab group (*n* = 198)	Zoledronic acid group (*n* = 215)	*P*-value
Adverse Reactions, n (%)			
Myalgia	2 (1.0)	6 (2.7)	
Dizziness	2 (1.0)	8 (3.7)	
Fever	3 (1.5)	10 (4.6)	
Bone pain	1 (0.5)	5 (2.3)	
Gastrointestinal	2 (1.0)	8 (3.7)	
Pruritus	1 (0.5)	6 (2.7)	
Total patients with adverse reactions	11 (5.56)	43 (20.00)	0.008
New fractures, *n* (%)	3 (1.5)	4 (1.9)	0.787

Adverse events were counted per patient. Incidence rates are presented for descriptive comparison only; no conclusions regarding differential safety superiority are drawn due to the passive, non-standardized nature of adverse event ascertainment.

The incidence of new fractures during the 36-month follow-up was low and not significantly different between the denosumab group (3/198, 1.5%) and the zoledronic acid group (4/215, 1.9%; *P* = 0.787) (Table 6).

## 4 Discussion

This 36-month retrospective study provides a head-to-head comparison of denosumab and zoledronic acid in a Chinese cohort with primary osteoporosis. Our findings demonstrate that while both agents are effective and well-tolerated treatments, in this cohort, denosumab was associated with significantly greater improvements in BMD at the lumbar spine, femoral neck, and total hip, more profound suppression of bone resorption (as reflected by β-CTX), greater pain relief, and a lower incidence of acute-phase reactions compared with zoledronic acid. These observations should not be interpreted as evidence of overall clinical superiority of denosumab over zoledronic acid.

The BMD gains observed with denosumab in this cohort are consistent with previous research, including a study by Kang et al., which reported greater lumbar spine BMD increases with denosumab compared to zoledronic acid in postmenopausal women (11). The absolute increases in lumbar spine BMD were 0.13 g/cm^2^ (18.1% change) in the denosumab group and 0.10 g/cm^2^ (13.9% change) in the zoledronic acid group. At the femoral neck, absolute increases were 0.14 g/cm^2^ (21.2% change) versus 0.12 g/cm^2^ (18.5% change), respectively. At the total hip, absolute increases were 0.09 g/cm^2^ (13.4% change) versus 0.05 g/cm^2^ (7.6% change), respectively. The significant improvement at the total hip, a site comprising both trabecular and cortical bone, underscores denosumab’s potent systemic anti-resorptive effect. The substantial increase at the femoral neck, a critical site for hip fracture, suggests a potential advantage in preventing this devastating complication. These findings support the clinical utility of denosumab, particularly in patients for whom maximizing BMD is a primary goal.

The BTM results provide mechanistic insight into these observations. The significantly greater reduction in β-CTX, a specific marker of bone resorption, confirms denosumab’s more potent inhibitory effect on osteoclast activity. The concurrent decrease in P1NP, a bone formation marker, in both groups reflects the physiological coupling of bone formation to resorption, with the magnitude of the P1NP reduction mirroring the degree of resorption inhibition. Denosumab’s more profound suppression of bone turnover observed in this study is likely a key driver of its greater BMD gains in this cohort.

Clinically, both treatments led to substantial improvements in pain and function, highlighting their value in enhancing patient quality of life. The greater pain reduction with denosumab may be attributable to its rapid and potent inhibition of bone resorption, which can alleviate microfractures and associated bone pain (2). However, as this study did not include imaging-based confirmation, we cannot attribute this effect specifically to reduced microfractures. Given that VAS scores in this study primarily reflected osteoporosis-related bone pain (including resting pain and activity-exacerbated pain in the lumbosacral spine and hip regions), the observed improvements likely represent a combination of analgesic and antiresorptive effects. This analgesic benefit could be an important consideration in treatment selection for patients with significant pain.

In addition to the efficacy and safety profiles, the long-term management strategies for denosumab and zoledronic acid present distinct clinical challenges that merit careful consideration. Unlike zoledronic acid, which has a prolonged skeletal residence time and a residual effect after discontinuation, denosumab’s effects are reversible and rapidly diminish upon cessation (19). This pharmacological difference imposes a critical requirement for rigorous adherence and a well-planned transition strategy. A study on personalizing denosumab therapy in postmenopausal Indian women with osteoporosis highlighted the predictive role of bone turnover markers and body mass index in determining the dosing interval, suggesting that tailored regimens based on these biomarkers could optimize clinical outcomes (20). This personalized approach is particularly relevant in Asian populations, where unique demographic and anthropometric characteristics may influence drug response. However, the most pressing concern with denosumab therapy is the risk of rebound vertebral fractures following its discontinuation, a phenomenon that has been increasingly recognized as a significant clinical issue. As emphasized by Jha et al., the abrupt withdrawal of denosumab without appropriate sequential therapy can lead to a rapid increase in bone resorption, surpassing pre-treatment levels and precipitating multiple vertebral fractures (21). This risk underscores the absolute necessity of transitioning patients who discontinue denosumab to a potent bisphosphonate, such as zoledronic acid, to prevent rapid bone loss and the consequent fracture risk. The absence of such a transition plan in our retrospective cohort, where denosumab was discontinued after 36 months without mandated follow-up therapy, represents a potential limitation and highlights an area for clinical quality improvement. These long-term management considerations—patient adherence, personalized dosing, and mandatory sequential therapy—are crucial for optimizing the risk-benefit profile of denosumab and should be integral to clinical decision-making, particularly in Asian populations where data on long-term outcomes are still accumulating.

The safety findings are noteworthy. The lower incidence of acute-phase reactions, such as fever and myalgia, with denosumab is consistent with its mechanism of action as a targeted biological agent, avoiding the cytokine release associated with intravenous bisphosphonate infusion (22). However, we refrain from concluding that denosumab has a superior overall safety profile, as the adverse event data were collected via passive retrieval from electronic medical records and may be subject to underreporting. The incidence rates of specific adverse events are presented descriptively in Table 6. Importantly, no serious adverse events, including osteonecrosis of the jaw or atypical femoral fractures, were observed in either group, consistent with the generally good safety profile of these agents when used appropriately.

The low and comparable rates of new fractures in both groups (1.5% and 1.9%) provide descriptive information on fracture occurrence in this cohort. However, the lack of a significant difference in fracture risk reduction between groups must be interpreted with caution. This may be attributable to the relatively short follow-up duration and modest sample size of our study, which may have been underpowered to detect differences in fracture outcomes. Landmark trials such as FREEDOM (denosumab) and HORIZON-PFT (zoledronic acid) required larger populations and longer observation periods to demonstrate significant fracture risk reduction (19). While BMD improvements are strongly correlated with fracture risk reduction, directly demonstrating a differential effect on fractures often requires larger-scale, longer-term studies. Fractures in this study were identified through patient self-report during telephone and outpatient follow-up; therefore, our fracture data should be considered descriptive and hypothesis-generating rather than definitive. Thus, denosumab’s potential for fracture prevention suggested by its BMD benefit requires confirmation in future prospective studies with fracture as the primary endpoint.

This study has several strengths. To our knowledge, it is one of the few long-term (36-month), head-to-head comparisons of denosumab and zoledronic acid specifically in a Chinese population, addressing an important evidence gap. The comprehensive assessment of outcomes, including multi-site BMD, BTMs, patient-reported outcomes, and safety, provides a holistic view of treatment effects. The real-world setting enhances the generalizability of our findings to routine clinical practice.

However, several limitations must be acknowledged. The retrospective, single-center design is susceptible to selection bias and unmeasured confounding. The 36-month follow-up, while substantial, may be insufficient to fully assess long-term efficacy, particularly regarding rare adverse events and differential effects on fracture risk. Medication adherence was not quantitatively assessed. Although the proportion of missing data was small, our complete-case analysis may still introduce some degree of bias. Finally, the sample size, while reasonable for comparative efficacy, was not powered to detect statistically significant differences in fracture rates, a relatively infrequent outcome; fracture data should therefore be viewed as exploratory. Future multi-center, prospective, randomized controlled trials with longer follow-up periods and fracture as the primary endpoint are warranted to validate and extend our findings.

In conclusion, this 36-month retrospective study demonstrates that both denosumab and zoledronic acid are effective and safe treatments for primary osteoporosis in a Chinese population. However, denosumab was associated with greater improvements in BMD at multiple skeletal sites, more potent suppression of bone resorption, pain relief, and a lower incidence of adverse reactions compared to zoledronic acid. These findings suggest that denosumab may be a particularly valuable treatment option for patients seeking maximal BMD gains, those with significant bone pain, or individuals intolerant to bisphosphonates. Given the retrospective, single-center, and non-randomized design, all findings must be regarded as tentative and hypothesis-generating. The statistical significance reported is descriptive and may be subject to confounding and bias. Therefore, these results are not sufficient to support definitive conclusions about treatment selection or overall clinical superiority of either agent, and require independent validation in well-designed prospective trials with fracture as the primary endpoint.

## Data Availability

The datasets presented in this study can be found in online repositories. The names of the repository/repositories and accession number(s) can be found in the article/supplementary material.
